# Providing organic macro minerals and an elevated platform improved tibia characteristics, and increased locomotion and performance of fast- and slower-growing broilers

**DOI:** 10.1016/j.psj.2022.101973

**Published:** 2022-05-21

**Authors:** Jerine A.J. van der Eijk, Jeroen Bakker, Bahadir C. Güz, Marinus M. van Krimpen, Roos Molenaar, Henry van den Brand, Ingrid C. de Jong

**Affiliations:** ⁎ Wageningen Livestock Research, Wageningen University and Research, PO Box 338, 6700 AH Wageningen, The Netherlands; † Adaptation Physiology Group, Wageningen University and Research, PO Box 338, 6700 AH Wageningen, The Netherlands

**Keywords:** organic macro minerals, enrichment, leg health, behavior, broiler

## Abstract

Improving leg health will support broiler health and welfare. Known factors to improve leg health are: replacing inorganic by organic macro minerals in the diet, providing environmental enrichments and using slower-growing broilers. However, it remains unknown how fast- and slower-growing broilers respond to a combination of providing organic macro minerals and an elevated platform as enrichment with regard to leg health. Therefore, the aim of this study was to identify whether a combined treatment of organic macro minerals and a platform affected leg health, tibia characteristics, behavior and performance of fast- and slower-growing broilers in a semicommercial setting. The experiment had a 2 × 2 factorial arrangement, with 12.800 fast-growing (Ross 308) and 12.800 slower-growing (Hubbard JA757) broilers that were randomly allocated to a control (i.e., inorganic macro minerals without enrichment) or adapted treatment (i.e., organic macro minerals and a platform). Broilers were housed in groups of 800 per pen (47.5 m^2^), with 8 replicates per treatment (total of 32 pens). Performance was measured weekly and over the total rearing period. Behavior was observed via scan sampling at a target weight of 0.6 and 1.9 kg for both breeds. Walking ability (gait score), footpad dermatitis, and hock burn were assessed in 10 broilers per pen just prior to slaughter weight. Leg disorders and tibia characteristics were assessed in the same broilers at slaughter weight (2.3 kg). Hardly any interaction effects between breed and treatment were found on leg health, tibia characteristics, behavior or performance, suggesting fast- and slower-growing broilers responded to the treatment similarly. The adapted treatment improved tibia characteristics, and increased locomotion and performance, but did not affect leg disorders, walking ability or contact dermatitis in both fast- and slower-growing broilers. The positive effects of the adapted treatment on tibia characteristics in both fast- and slower-growing broilers may improve leg health, although the current study did not confirm this for leg disorders, walking ability or contact dermatitis.

## INTRODUCTION

Genetic selection and improved rearing conditions have caused broilers to grow to slaughter weight in a short period of time (Zuidhof et al., 2014). This efficient growth has led to welfare problems in broilers, including leg disorders and bone deformations, causing leg weakness, impaired walking ability and extended periods spent sitting or lying, which can further cause contact dermatitis on feet and hocks (Bradshaw et al., 2002; Bessei, 2006; Knowles et al., 2008; EFSA, 2010; Tahamtani et al., 2018). As a consequence, broilers can experience difficulties to perform natural behaviors and to access feed and water, and may suffer from pain and discomfort (McGeown et al., 1999; Danbury et al., 2000; Weeks et al., 2000; Bradshaw et al., 2002; Caplen et al., 2013; Hothersall et al., 2016). Impaired leg health is considered to be one of the most important factors affecting health and welfare of broilers (EFSA, 2010; Hartcher and Lum, 2020; de Jong, 2020), where leg health includes lameness (i.e., infectious, degenerative and developmental disorders) and contact dermatitis on the feet and hocks (i.e., footpad dermatitis and hock burn) (Bradshaw et al., 2002).

Impaired leg health has been associated with deficiencies and excesses of vitamins, minerals, proteins, amino acids and fatty acids. Nutrients of major concern for bone development are vitamin D, calcium (Ca), and phosphorus (P) (Oviedo-Rondon et al., 2006). Replacing inorganic by organic minerals has been shown to improve their intestinal absorption and bioavailability (Bao et al., 2007, 2010; Nollet et al., 2007), thereby it could improve bone mineralization and quality. Previous studies have shown that replacing inorganic with organic macro (Ca and P) and trace (Fe, Cu, Mn, Zn and Se) minerals in the diet improved tibia characteristics in fast-growing broilers (Güz et al., 2019). These positive effects were most likely caused by the organic macro minerals, since providing organic trace minerals alone did not affect tibia characteristics in fast-growing broilers (Güz et al., 2021b). Thus, replacing inorganic with organic macro minerals seems to improve bone quality and could therefore improve leg health in broilers.

Impaired leg health is often associated with low levels of activity and it has been suggested that increasing locomotor activity of broilers might improve leg health (Kestin et al., 1992; Prayitno et al., 1997a,b; Reiter and Bessei, 2009; Stojcic and Bessei, 2009). Locomotor activity can be stimulated by providing environmental enrichments (Riber et al., 2018). Previous studies have shown that providing enrichments, such as barriers, perches, platforms or increasing distance between feeders and drinkers, improved walking ability in fast-growing broilers (Kaukonen et al., 2017a; Vasdal et al., 2019), although these effects are not always found (de Jong and Gunnink, 2019; de Jong et al., 2021; Güz et al., 2021a) which might depend on the type of enrichments provided. Additionally, providing a combination of enrichments improved tibia characteristics in fast- and slower-growing broilers (Güz et al., 2021a). Environmental enrichments such as platforms or perches could further reduce the risk of contact dermatitis (Ventura et al., 2010), as they reduce contact with the litter, but also these effects are not always found (Pedersen and Forkman, 2019). Thus, providing environmental enrichments could improve tibia characteristics and leg health in broilers.

Although factors such as nutrition, management, and diseases play a role in the development of leg problems, growth rate seems to be the main influencer (Knowles et al., 2008). Slower-growing broiler breeds are increasingly used for meat production, particularly in Western-Europe (Vissers et al., 2019). These breeds need more time to reach slaughter weight compared to fast-growing broiler breeds. Therefore, leg bones have a longer time to develop, resulting in more mature and robust bones (Stojcic and Bessei, 2009). Consequently, weight load seems to have less impact on slower-growing broilers and it is suggested this results in a better walking ability and increased locomotor activity compared to fast-growing broilers (Bizeray et al., 2000; Reiter and Bessei, 2009; Stojcic and Bessei, 2009; Vasdal et al., 2019). Slower-growing broilers are therefore also suggested to make better use of environmental enrichments (Bokkers and Koene, 2003; Malchow et al., 2019; Rayner et al., 2020), which could further improve leg health as mentioned previously. Thus, slower-growing broilers seem to have better leg health compared to fast-growing broilers.

Overall, replacing inorganic with organic macro minerals, providing environmental enrichments and using slower-growing broilers might improve leg health and tibia characteristics. For fast-growing broilers, organic macro minerals improved tibia characteristics (Güz et al., 2019, 2021b), and for fast- and slower-growing broilers, environmental enrichment had a positive effect on tibia characteristics (Güz et al., 2021a). However, it remains unknown whether the combination of organic macro minerals and environmental enrichments has an additive positive effect on leg health and tibia characteristics. Therefore, the aim of this study was to identify the combined effect of providing organic macro minerals and environmental enrichments on leg health, tibia characteristics, behavior and performance of fast- and slower-growing broilers in a semicommercial setting. The diet consisted of replacing inorganic with organic macro minerals, as this was previously shown to positively affect tibia characteristics (Güz et al., 2019, 2021b). As the combination of enrichments that improved tibia characteristics previously (Güz et al., 2021a) are not easily applied in a commercial setting, a potentially successful environmental enrichment for improving walking ability of broilers was selected and included a platform with ramps (Norring et al., 2016; Kaukonen et al., 2017a; Vasdal et al., 2019). We hypothesized that the combined treatment would improve leg health and tibia characteristics in both fast- and slower-growing broilers.

## MATERIALS AND METHODS

### Experimental Design

The experiment had a 2 × 2 factorial design with 2 broiler breeds, fast-growing (Ross 308, **FAST**) or slower-growing (Hubbard JA757, **SLOW**) broilers that had access to a control diet with inorganic macro minerals and nonenriched environment (control, **CON**) or to a diet with organic macro minerals and enriched environment (adapted, **ADAP**). The experiment was carried out in a semicommercial environment at Schothorst Feed Research (Lelystad, The Netherlands). The experiment was approved by the Central Authority for Scientific Procedures on Animals in accordance with the Dutch regulations (no: AVD401002016686).

### Animals, Housing, and Diets

Day-old broiler chicks, originating from a parent stock of 40 (FAST) and 45 (SLOW) wk of age, were obtained from a commercial hatchery (Probroed, Groenlo, The Netherlands). A total of 12.800 FAST and 12.800 SLOW broilers (as hatched) were randomly allocated to the CON or ADAP treatment, resulting in 4 experimental groups (FAST-CON, FAST-ADAP, SLOW-CON, and SLOW-ADAP). Each experimental group was replicated 8 times, with a total of 32 experimental pens divided over 2 rooms (16 pens per room) in one house. Each experimental group was equally assigned to the 2 rooms with 4 replicates per room. One SLOW-ADAP pen did not receive the environmental enrichment and was therefore excluded from the experiment. A complete randomized block design was used, that is, experimental groups were randomly distributed within a block of 4 pens. Pen was the experimental unit and each pen contained 800 broilers.

Both rooms were identical and climate controlled with a temperature of 33°C at arrival, which gradually decreased to a constant temperature of 18°C at 40 d of age. The lighting program used was 24L:0D at arrival, 20L:4D from d 1 to 6 and 18L:6D from d 7 onward. Light intensity at chick height (±25 cm) was on average 36.9 lux and ranged between 30.4 and 47.6 lux. Floor pens (47.5 m^2^, length 9.5 m, width 5 m, and height 0.75 m) had wood shavings as litter and further included 11 pan feeders and 72 nipple drinkers with cups. For the ADAP treatment, pens included an enrichment in the form of a plastic wire mesh platform (length 200 cm, width 100 cm, and height 40 cm) equipped with 2 ramps of the same material (each with length 100 cm, width 100 cm, and angle of 12°) that was positioned between the feeding and drinking line. Four firmly pressed straw bales were used to support the platform (length 50 cm, width 30 cm, and height 40 cm). For SLOW broilers, pens included a net up to 1.6 m high to avoid them from escaping to other pens.

Broilers had ad libitum access to feed and water. A three-phase feeding schedule was applied with a starter diet (d 0 to 14), grower diet (d 14 to 35), and finisher diet (d 35 onward). For the ADAP treatment, inorganic macro minerals were replaced by organic macro (Ca, P) minerals without changing the mineral level. The inorganic macro minerals Ca and P, provided by limestone and monocalcium phosphate were replaced by Calfos (Sonac Vuren B.V., Vuren, The Netherlands), an organic Ca and P source originating from processed porcine bones in which the Ca and P are embedded in collagen and where the original hydroxyapatite structure has been preserved. For Ca this was done for 72% in the starter diet, 53% in the grower diet, and 26% in the finisher diet. For P this was done for 100% in all diets. All diets were produced and pelleted by ABZ Diervoeding (Leusden, The Netherlands) and analyzed for crude ash (ISO 5984), crude protein (ISO 5983), crude fat (ISO 6492), and crude fiber (ISO 6865) using the Weende (proximate) analysis, and Ca (ISO 6869) and P (ISO 6941) by NutriControl (Veghel, The Netherlands). Diet compositions, calculated and analyzed nutrient values are shown in Table 1. Chicks received Aviguard (probiotic mixture) and were vaccinated against Infectious Bronchitis at the hatchery. Chicks were further vaccinated against Newcastle Disease via spray at 8 d of age and against Infectious Bursal Disease plus Gumboro via the drinking water at 21 d of age.

**Table 1:** (A) Ingredients (%), (B) calculated, and (C) analyzed nutrients of the experimental diet (g/kg, as-fed basis); CON = control (i.e., inorganic macro minerals without enrichment), ADAP = adapted treatment (i.e., organic macro minerals and a platform).

AIngredients (%)	Starter (0–14 d)			Grower (14–35 d)				Finisher (35–51 d)	
	CON	ADAP		CON	ADAP			CON	ADAP
Corn	35.00	35.00		30.00	30.00			25.00	25.00
Wheat	24.38	24.38		35.02	35.02			43.81	43.81
Soybean meal (>48% CP)	26.69	26.69		17.66	17.66			12.72	12.72
Sunflower meal (37% CP)	2.72	2.72		4.00	4.00			5.00	5.00
Rape seed meal	0.00	0.00		2.00	2.00			3.00	3.00
Oat hulls	2.00	2.00		2.00	2.00			2.00	2.00
Wheat middling	0.00	0.00		0.73	0.73			0.89	0.89
Soybean oil	1.54	1.54		1.06	1.06			1.25	1.25
Animal fat - Poultry	2.92	2.92		3.39	3.39			3.40	3.40
Salt	0.09	0.09		0.07	0.07			0.05	0.05
Lysine HCL (79%)	0.30	0.30		0.26	0.26			0.31	0.31
Methionine L/DL (99%)	0.25	0.25		0.18	0.18			0.15	0.15
Threonine L (98%)	0.08	0.08		0.07	0.07			0.08	0.08
Tryptophan L (98%)	0.00	0.00		—	—			—	—
Valine L (99%)	0.02	0.02		—	—			—	—
P premix Sacox 2314	—	—		0.58	0.58			—	—
P premix Maxiban +	0.50	0.50		—	—			—	—
Xylanase-glucanase premix	0.25	0.25		0.25	0.25			0.25	0.25
Sodium bicarbonate	0.43	0.43		0.38	0.38			0.41	0.41
Vitamin and mineral premix	0.50	0.50		0.50	0.50			0.50	0.50
Phytase	0.01	0.01		0.01	0.01			0.01	0.01
Monocalcium phosphate	0.90	—		0.55	—			0.20	—
Calfos^1^	—	1.59		—	0.97			—	0.34
Limestone	1.44	0.51		1.28	0.71			0.99	0.79
Diamol	—	0.24		—	0.15			—	0.05
Total	100	100		100	100			100	100


B	Starter (0–14 d)			Grower (14–35 d)			Finisher (35–51 d)		
Calculated nutrients (g/kg)	CON		ADAP	CON		ADAP	CON		ADAP
Moisture	116.51		117.14	117.27		117.66	117.57		117.71
Crude ash	55.89		52.68	47.03		45.06	39.21		38.51
Crude protein	200.00		201.59	176.28		177.26	165.88		166.22
Crude fat	71.25		71.73	70.64		70.93	72.14		72.24
Crude fiber	30.21		30.21	33.50		33.50	35.40		35.40
Ca	8.30		8.30	6.80		6.80	5.10		5.10
P	5.58		5.69	4.83		4.90	4.06		4.08
Mg	1.60		1.63	1.59		1.60	1.57		1.57
K	8.54		8.57	7.37		7.39	6.68		6.68
Na	1.60		1.70	1.40		1.46	1.40		1.42
Cl	1.70		1.70	1.50		1.50	1.50		1.50
Electrolyte balance (mEq)	240		244	208		210	190		191
Retainable P	4.40		4.40	3.70		3.70	3.00		3.00
AME^2^ (kcal/kg)	2,982		2,989	3,011		3,015	3,048		3,049
AFD^2^ lysine	11.10		11.17	9.10		9.14	8.60		8.61
AFD^2^ methionine	5.22		5.24	4.31		4.32	3.90		3.90
AFD^2^ cysteine	2.68		2.68	2.52		2.52	2.47		2.47
AFD^2^ met+cys	7.88		7.90	6.83		6.84	6.36		6.37
AFD^2^ threonine	6.88		6.93	5.92		5.94	5.59		5.60
AFD^2^ tryptophan	2.11		2.12	1.82		1.82	1.70		1.70
SID^2^ lysine	11.21		11.27	9.17		9.21	8.64		8.65
SID^2^ methionine	5.27		5.29	4.36		4.37	3.95		3.96
SID^2^ cysteine	2.62		2.62	2.52		2.52	2.49		2.49
SID^2^ met+cys	7.93		7.95	6.91		6.92	6.46		6.47
SID^2^ threonine	6.81		6.85	5.88		5.91	5.57		5.58
SID^2^ tryptophan	2.07		2.07	1.80		1.80	1.69		1.69

C	Starter (0–14 d)			Grower (14–35 d)			Finisher (35–51 d)		
Analyzed nutrients (g/kg)	CON		ADAP	CON		ADAP	CON		ADAP
Crude ash	53		56	49		46	40		38
Crude protein	210		208	181		180	169		171
Crude fat	65		62	62		62	66		64
Crude fiber	32		32	34		33	39		40
Ca	7.95		8.64	7.88		6.12	5.78		4.65
P	5.43		6.22	5.33		4.97	4.38		4.05

1 Composition of Calfos provided per kg of product: 100 g crude protein, 300 g calcium, 130 g phosphorus (113 g digestible phosphorus), 50 g moisture.

2 Abbreviations: AFD, apparent fecal digestibility; AME, apparent metabolizable energy; SID, standardized ileal digestibility.

### Performance

Body weight (**BW**) at pen level was measured using an automatic weighing plateau, to which broilers had voluntary access. Slaughter weight was based on container weights at the end of the trial. Feed intake (**FI**) and feed conversion ratio (**FCR**) were determined for the total rearing period and corrected for the weight at mortality (**MRT**) as previously shown by Dersjant-Li et al. (2013) with slight modifications. FCR was calculated using the following formula:

$$FCR\;in\;period\;x-y=\;\frac{total\;feed\;intake\;in\;period\;x-y}{\left(total\;live\;weight+weight\;dead\;birds\right)y-\left(total\;live\;weight\right)x}$$

Weight of dead birds was calculated by taking the number of dead birds on day x * 0.8 * average weight on day x based on the weighing plateaus. The factor 0.8 was used to account for the generally lower body weight of weak(er) birds that have a higher likelihood to die. MRT was noted daily at pen level. Litter quality was weekly scored visually at pen level on a scale of 0 to 10 according to Dersjant-Li et al. (2015), where litter score (LTS) 0 corresponded with low litter quality (wet, caked) and score 10 corresponded with high litter quality (dry, friable).

### Behavioral Observations

Behavior was observed at pen level using instantaneous scan sampling at 2 ages (d 17 and 34 for FAST and d 20 and 48 for SLOW). These ages were chosen based on similar target weights of FAST and SLOW broilers (0.6 kg at young age and 1.9 kg at older age). Actual weights during observations slightly differed from target weights with FAST having an average BW of 0.6 and 1.8 kg, and SLOW of 0.5 and 2.0 kg, respectively. Each pen was observed once in the morning (08:30–13:00) and once in the afternoon (13:00–17:30) on each observation day. Each observation consisted of scoring a fixed area within the pen (±6 m^2^) which was scanned 5 times after a 5 min habituation period. Per scan, the behavior of all broilers in the area was scored according to the ethogram in Table 2. Behavioral observations were performed by 2 observers. Reliability between the 2 observers (interobserver agreement) was high (index of concordance: 0.74). For the ADAP treatment, the use of the enrichment was scored by counting the number of chickens on or under the platforms (including ramps).

**Table 2:** Ethogram used for behavioral observations.

Behavior	Description
Eating	Having the head above or in the feeder or pecking at feed in the feeder
Drinking	Pecking at the drinking nipples or cup beneath the drinking nipple
Inactive	Sitting or lying while not engaged in any other activities
Locomotion	Walking, running, jumping or hopping without performing any other type of behaviour
Standing	Standing without performing any other type of behavior
Ground pecking	Inactive and pecking at the ground, litter
Foraging	Pecking and/or scratching at the ground, litter
Comfort	Preening (manipulating own feathers with the beak or paws), stretching, wing flaps, feather ruffles, shakes (outside context of dust bathing)
Dustbathing	Rubs head and body against the ground, pecks and scratches while lying on the side, distributes substrate over body or shakes off substrates from feathers
Aggressive behavior	All elements of aggressive behavior, such as hopping oriented towards another chicken, threatening (both upright position), leaping, kicking, wing flapping or aggressive pecking (pecking directed to the head)
Other	All other behaviors not described above

### Gait Score, Footpad Dermatitis, and Hock Burn

Gait score (**GS**), footpad dermatitis (**FPD**), and hock burn (**HB**) were assessed at individual level (n = 10 per pen, including 5 males and 5 females) on d 36 for FAST and d 49 of age for SLOW broilers. Individuals were selected that had a BW close to the average BW for males and females per breed based on pen weights, with an average BW of 2.201 and 2.242 kg, for FAST and SLOW broilers, respectively. GS, FDP and HB were assessed according to the Welfare Quality protocol (Welfare Quality®, 2009) by one trained observer.

### Leg Disorders and Breast Myopathies

Leg disorders and breast myopathies, that is, wooden breast (**WB**) and white striping (**WS**), were assessed at individual level (n = 10 per pen) on d 37 for FAST and d 50 of age for SLOW broilers. Previously selected individuals for GS, FPD and HB were slaughtered by electrocution at an average BW of 2.303 and 2.271 kg, for FAST and SLOW broilers, respectively. After fixating the legs at the hip joint to stretch the leg, both legs were scored for varus-valgus (**VV**) by determining the angle between the tibia and the metatarsus using a goniometer. The left leg of each chicken was assessed by a veterinarian and scored on occurrence of leg disorders, including tibia dyschondroplasia (**TD**), bacterial chrondronecrosis with osteomyelitis (**BCO**), epiphyseal plate abnormalities (**EPA**), and epiphyseolysis (**EPI**). All abnormalities were scored as0 (no abnormalities) or 1 (abnormalities). Breast myopathies were assessed by one observer and scored on a 0 (normal) to 3 (extreme) scale according to Kuttappan et al. (2016).

### Tibia Characteristics

The right leg of each chicken was collected, defleshed and tibias were obtained and stored until further analyses at -20°C. The whole bone was used for all measurements of tibia characteristics. After thawing, tibia weight, length, thickness, femoral head thickness, metatarsal head thickness, osseous volume, pore volume, total volume (osseous volume + pore volume), volume fraction (osseous volume/total volume), mineral content, and mineral density were analyzed at individual level (n = 6 per pen, including 3 males and 3 females) using a GE Phoenix 3D X-ray microfocus CT scanner (General Electric Company, Boston, MA) as described by Bouxsein et al. (2010) and Güz et al. (2021a). Robusticity index was calculated, using the following formula: robusticity index (cm/g) = bone proximal length (cm)/bone weight (g) as described by (Seedor et al., 1991). Where bone proximal length was determined by measuring the 2 end-points of the bone.

Tibias were subjected to a three-point bending test as described by Jungmann et al. (2007), using an Instron universal testing machine (Instron, Norwood, MA). Ultimate stress (maximal load of breaking point) data was used for tibia ultimate strength; the slope of the selected linear part of the curve data was used as the tibia stiffness; the area under the curve of selected region data was used as the tibia energy to fracture. Elastic modulus (**GPa**) was calculated with the following formula as described by Turner and Burr (1993):

$$E\;=\;N\;\;\;S^{3}/4\delta \;\;\;TL^{3}$$

where E is the elastic modulus (GPa), N is the maximal load (N), S is the span between bending fixtures (mm), T is the tibia thickness (mm), L is the tibia length (mm), and δ is the maximum deflection (mm) at the midpoint of the bone.

### Statistical Analysis

GenStat version 19.1 (VSN International, Hemel Hempstead, UK) was used for statistical analysis of body weight development at pen level on logscale, using a random regression model with a 3rd polynomial model (1):

(1) $${\underline{Y}}_{ijkl}=\left(\beta 0_{ij}+\underline{\varepsilon }0_{ijk}\right)+\left(\beta 1_{ij}+\underline{\varepsilon }1_{ijk}\right)*X+\left(\beta 2_{ij}+\underline{\varepsilon }2_{ijk}\right)*X^{2}+\left(\beta 3_{ij}+\underline{\varepsilon }3_{ijk}\right)*X^{3}+{\underline{\varepsilon }}_{ijkl}$$

with Y = dependent variable, *β0* = intercept, *β1* = linear, *β2* = quadratic, *β3* = cubic, X = age (weeks) and Ԑ_ijkl_ = residual error term, _i_ = breed, _j_ = treatment, _k_ = pen, and _l_ = weight measure-number within pen. For model simplification, nonsignificant (*P* > 0.1) terms from model [1] were removed, which included the interaction terms between breed and treatment, and quadratic and cubic terms for treatment.

SAS Software version 9.4 was used for further statistical analysis (SAS Institute Inc., Cary, NC) and data were analyzed at pen level. Normality of the data was assessed based on model residuals. BW and LTS were analyzed per week, and FI, FCR, and MRT were analyzed for the total rearing period using a MIXED-procedure with model (2):

(2) $$\underline{Y}\;=\;\mu \;+\;breed\;+\;treatment\;+\;breed\;*\;treatment\;\;+\underline{\varepsilon }$$

with Y = dependent variable, *µ* = overall mean, breed = fixed effect of breed (FAST or SLOW), treatment = fixed effect of treatment (CON or ADAP), breed*treatment = interaction between breed and treatment, and Ԑ = residual error term. Block (1 to 8) was included as random effect.

Behavioral data was aggregated and expressed as percentage of broilers performing a certain behavioral category: ingestion (eating and drinking), active, inactive (inactive and standing), comfort (comfort and dust bathing), foraging (foraging and ground pecking). Behavior was analyzed per target weight (0.6 and 1.9 kg) using a MIXED-procedure with model (3):

(3) $$\underline{Y}\;=\;\mu \;+\;breed\;+\;treatment\;+\;breed\;*\;treatment\;+\;time\;+\;observer\;+\underline{\varepsilon }$$

with time = fixed effect of time (morning or afternoon) and observer = fixed effect of observer (1 or 2). Pen (1–32) within breed and treatment, and block (1–8) were included as separate random effects. Occurrences of aggressive and other behavior were very low, therefore these behaviors were analyzed with model [3] using a GLIMMIX-procedure with a binary distribution (0 vs. > 0). For enrichment use, calculations were made to obtain percentages of broilers using the enrichment. Enrichment use was analyzed per target weight (0.6 and 1.9 kg) using a MIXED-procedure with model [3], but without the fixed effects of treatment and breed*treatment interaction. Pen (1–32) within block (1–8) was included as random effect.

GS, FPD, and HB were analyzed using a GLIMMIX-procedure with a multinomial distribution with model [4]. Leg disorders (EPA and EPI) and WB were analyzed using a GLIMMIX-procedure with a binary distribution (0 vs. >0) with model (4). Occurrences of TD, BCO and WS were very low (n = 2, n = 7, and n = 16 birds with score >0, respectively), therefore these variables were excluded from statistical analysis.

(4) $$\underline{Y}\;=\;\mu \;+\;breed\;+\;treatment\;+\;breed\;*\;treatment\;+\;sex\;+\underline{\varepsilon }$$

Pen (1–32) within breed and treatment, and block (1–8) were included as separate random effects. VV was analyzed using a MIXED-procedure with model [4] where the fixed effect of side (left or right) was further included. Tibia characteristics were analyzed using a MIXED-procedure with model [4]. BW was added to model [4] as a covariable for GS, FPD, HB (BW of day prior to slaughter) and for EPA, EPI, WB, VV, and tibia characteristics (BW at slaughter). Post hoc pairwise comparisons were corrected by Tukey-Kramer adjustment. Data are presented as LSmeans ± pooled standard error of the mean (SEM) for the MIXED procedure and as means ± pooled SEM for the GLIMMIX procedure, unless otherwise mentioned.

## RESULTS

### Performance

**Figure 1:**
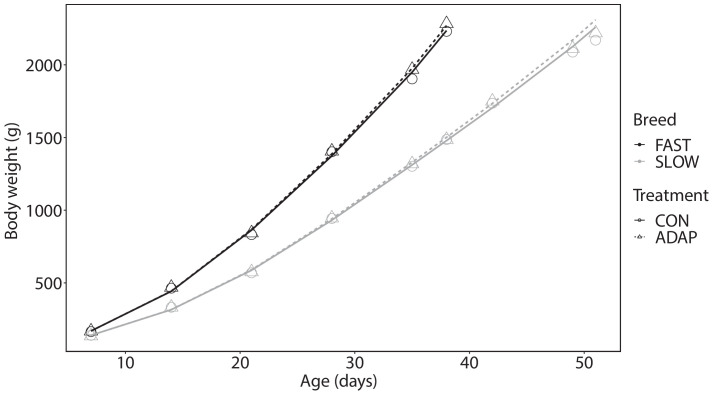
Body weight development, symbols represent untransformed means, lines represent model fits for fast- (FAST, black) and slower-growing broilers (SLOW, grey) in the control (CON, circles, solid line) or adapted treatment (ADAP, triangles, dashed line) (n = 8 pens for FAST-CON, FAST-ADAP and SLOW-CON, n = 7 pens for SLOW-ADAP). From 38 d of age onward only slower-growing broilers were in the experiment.

For BW development there was no significant interaction effect between breed and treatment. However, breeds significantly differed (linear, quadratic and cubic) with FAST broilers having a higher BW compared to SLOW broilers over time (*P* < 0.001). Furthermore, treatments significantly differed (linear, but not quadratic and cubic), indicating that broilers in ADAP treatment had a higher body weight compared to broilers in CON treatment over time (*P* = 0.023) (Figure 1).

Other performance results are summarized in Table 3. No interaction effects were found for any of the performance characteristics, except for ABW on d 35. FAST broilers in the ADAP treatment had higher ABW compared to other groups, but SLOW broilers in CON or ADAP treatment did not differ. Breed effects were found for all characteristics. ABW (except on d 0), slaughter weight, ADFI, and MRT were higher for FAST than for SLOW broilers. FCR and LTS were lower (better for FCR, worse for LTS) for FAST than for SLOW broilers. Treatment effects were found, with ABW (d 21, 35, and 51), slaughter weight, and ADFI being higher for broilers in ADAP treatment than for broilers in CON treatment.

**Table 3:** Effects of breed, treatment, and their interaction on performance characteristics.

Variable		Breed			Treatment			Interaction					*P*-values		
		FAST	SLOW	SEM	CON	ADAP	SEM	FAST-CON	FAST-ADAP	SLOW-CON	SLOW-ADAP	SEM	Breed	Treatment	Breed*Treatment
ABW^1^	D 0	43	42												
	D 7	166^a^	137^b^	1	152	151	1	165	168	139	135	2	<0.001	0.76	0.053
	D 14	466^a^	332^b^	2	398	400	2	464	469	332	332	2	<0.001	0.27	0.20
	D 21	838^a^	572^b^	3	701^b^	709^a^	3	832	844	569	575	4	<0.001	**0.02**	0.40
	D 28	1404^a^	945^b^	9	1,173	1,177	9	1,402	1,407	944	946	11	<0.001	0.74	0.94
	D 35	1936^a^	1,311^b^	9	1,603^b^	1,644^a^	9	1,904^b^	1,969^a^	1,302^c^	1,319^c^	11	<0.001	<0.001	0.02
	D 38	2258^a^	1,486^b^	15	1,860	1,884	15	2,233	2,283	1,487	1,486	21	<0.001	0.25	0.24
	D 42		1,743		1,736	1,750	9							0.30	
	D 49		2,100		2,088	2,111	11							0.11	
	D 51		2,195		2,170^b^	2,220^a^	19							0.03	
	Slaughter weight^2^	2251^a^	2,209^b^	10	2,203^b^	2,257^a^	10	2,213	2,288	2,192	2,226	14	0.008	<0.001	0.15
	ADFI^3^	91.3^a^	79.1^b^	0.3	84.6^b^	85.8^a^	0.3	90.6	92.0	78.6	79.6	0.4	<0.001	0.003	0.61
	FCR^4^	1.57^b^	1.86^a^	0.01	1.73	1.71	0.01	1.59	1.56	1.87	1.86	0.01	<0.001	0.07	0.23
	MRT^5^	1.90^a^	1.36^b^	0.17	1.78	1.48	0.17	2.08	1.71	1.49	1.24	0.23	0.03	0.19	0.79
LTS^6^	D 14	6.3^b^	7.9^a^	0.1	7.0	7.1	0.1	6.3	6.3	7.8	8.0	0.2	<0.001	0.61	0.61
	D 21	5.8^b^	7.7^a^	0.1	6.7	6.8	0.1	5.6	5.9	7.8	7.6	0.2	<0.001	0.53	0.21
	D 28	5.1^b^	6.5^a^	0.1	5.8	5.8	0.1	5.0	5.2	6.6	6.4	0.2	<0.001	0.94	0.37
	D 35	5.4^b^	6.5^a^	0.2	5.9	6.0	0.2	5.4	5.4	6.4	6.6	0.2	<0.001	0.68	0.68
	D 42		6.0		6.0	6.0									
	D 49		5.0		5.0	5.0									

a-b Per factor, values in a row lacking a common superscript differ significantly (*P* < 0.05).

1 ABW = average body weight (g) based on plateau weighing.

2 Slaughter weight based on container weighing.

3 ADFI = average daily feed intake (g/day).

4 FCR = feed conversion ration.

5 MRT = mortality in %.

6 LTS = litter score, where 0 = low litter quality and 10 = high litter quality. Abbreviations: ADAP, adapted treatment (i.e., organic macro minerals and a platform); CON = control (i.e., inorganic macro minerals without enrichment); FAST, fast-growing broilers; SLOW, slower-growing broilers; (n = 8 pens for FAST-CON, FAST-ADAP, and SLOW-CON, n = 7 pens for SLOW-ADAP). From 38 d of age onward only SLOW broilers were in the experiment.

### Behavior and Enrichment Use

Behavior and enrichment use results are summarized in Table 4. An interaction between breed and treatment was found for foraging behavior at 1.9 kg. However, after correction for multiple comparisons, no significant differences between experimental groups were found for foraging behavior. Breed effects were found for ingestion, locomotion, foraging and aggressive behavior. At 0.6 kg, FAST broilers showed more ingestion, less locomotion, foraging, and aggressive behavior than SLOW broilers. At 1.9 kg, FAST broilers showed less locomotion than SLOW broilers. Treatment effects were found for locomotion at 1.9 kg, with broilers in ADAP treatment showing more locomotion compared to broilers in CON treatment.

**Table 4:** Effects of breed, treatment, and their interaction on behavior and enrichment use, values are expressed as percentage of broilers showing a behavior or making use of enrichments.

Behavior	Breed			Treatment			Interaction					*P*-values		
	FAST	SLOW	SEM	CON	ADAP	SEM	FAST-CON	FAST-ADAP	SLOW-CON	SLOW-ADAP	SEM	Breed	Treatment	Breed*Treatment
0.6 kg														
Ingestion^1^	24.7^a^	20.6^b^	1.2	21.0	24.2	1.2	22.7	26.7	19.4	21.7	1.8	0.03	0.08	0.64
Inactive^2^	55.9	53.7	1.3	56.2	53.4	1.3	58.0	53.9	54.5	52.9	1.8	0.23	0.14	0.50
Locomotion	3.9^b^	5.4^a^	0.3	4.7	4.6	0.3	4.1	3.7	5.3	5.6	0.5	0.002	0.90	0.51
Comfort^3^	10.3	11.4	0.6	11.2	10.5	0.6	10.5	10.1	11.9	10.9	0.8	0.19	0.42	0.74
Foraging^4^	4.8^b^	8.1^a^	0.6	6.5	6.4	0.6	4.5	5.0	8.3	7.9	0.8	<0.001	0.94	0.61
Aggressive	0.23^b^	0.76^a^	0.11	0.40	0.58	0.12	0.14	0.33	0.66	0.87	0.15	0.04	0.32	0.59
Other	0.17	0.11	0.08	0.07	0.21	0.03	0.12	0.21	0.02	0.21	0.11	0.88	0.29	0.29
Area on platform/ramp	4.3^b^	8.5^a^	0.5		6.2							<0.001		
Area under platform/ramp	1.7	1.5	0.2		1.6							0.54		
1.9 kg														
Ingestion^1^	15.8	17.3	1.2	17.5	15.7	1.2	17.2	14.5	17.7	17.0	1.6	0.37	0.31	0.52
Inactive^2^	70.0	66.3	1.4	68.4	67.9	1.4	71.2	68.9	65.5	67.0	1.9	0.06	0.84	0.34
Locomotion	3.1^b^	4.8^a^	0.3	3.3^b^	4.6^a^	0.3	2.3	3.9	4.3	5.3	0.5	<0.001	0.01	0.49
Comfort^3^	6.0	6.2	0.4	5.8	6.3	0.4	5.7	6.2	5.9	6.4	0.5	0.66	0.39	0.93
Foraging^4^	5.0	5.1	0.6	4.8	5.2	0.6	3.4	6.5	6.2	4.0	0.9	0.87	0.64	0.006
Aggressive	0.12	0.24	0.07	0.18	0.17	0.07	0.08	0.16	0.28	0.18	0.09	0.20	0.58	0.82
Other	0.02	0.09	0.05	0.04	0.06	0.04	0.04	0.00	0.05	0.13	0.05			
Area on platform/ramp	3.5^b^	5.0^a^	0.2		4.2							<0.001		
Area under platform/ramp	1.2^a^	0.6^b^	0.1		1.0							<0.001		

a-b Per factor, values in a row lacking a common superscript differ significantly (*P* < 0.05).

1 Sum of eating and drinking.

2 Sum of inactive and standing.

3 Sum of comfort and dustbathing.

4 Sum of foraging and ground pecking. Abbreviations: ADAP, adapted treatment (i.e., organic macro minerals and a platform); CON, control (i.e., inorganic macro minerals without enrichment); FAST, fast-growing broilers; SLOW, slower-growing broilers; (n = 8 pens for FAST-CON, FAST-ADAP, and SLOW-CON, n = 7 pens for SLOW-ADAP).

For enrichment use, breed effects were found for the percentage of broilers on and under the platform/ramp. At both 0.6 and 1.9kg, percentage of SLOW broilers on the platform/ramp was higher compared to FAST broilers. At 1.9 kg, the percentage of FAST broilers under the platform/ramp was higher compared to SLOW broilers.

### Gait score, Footpad Dermatitis, and Hock Burn

No significant effects of the interaction between breed and treatment or treatment alone were found for GS, FPD and HB. Breed effects were found, with FAST broilers having higher (worse) scores for GS, FPD and HB than SLOW broilers just prior to slaughter weight (Table 5).

**Table 5:** Effects of breed, treatment, and their interaction on gait score, footpad dermatitis and hock burn, values represent means and percentages of birds per score are given.

Variable	Breed			Treatment			Interaction					*P*-values		
	FAST	SLOW	SEM	CON	ADAP	SEM	FAST-CON	FAST-ADAP	SLOW-CON	SLOW-ADAP	SEM	Breed	Treatment	Breed*Treatment
GS^1^	2.96^a^	2.62^b^	0.04	2.81	2.78	0.05	3.00	2.91	2.61	2.63	0.06	<0.001	0.61	0.53
0	0	0.7		0	0.7		0	0	0	1.4				
1	0	0		0	0		0	0	0	0				
2	12.5	42.0		27.5	26.0		10.0	15.0	45.0	38.6				
3	79.4	51.3		64.4	67.3		80.0	78.8	48.8	54.3				
4	8.1	6.0		8.1	6.0		10.0	6.3	6.3	5.7				
5	0	0		0	0		0	0	0	0				
FPD^2^	2.46^a^	0.98^b^	0.10	1.64	1.86	0.12	2.48	2.45	0.80	1.19	0.14	<0.001	0.44	0.25
0	13.8	57.3		40.6	28.7		16.3	11.3	65.0	48.6				
1	2.5	10.0		5.6	6.7		1.3	3.8	10.0	10.0				
2	23.8	11.3		11.9	24.0		17.5	30.0	6.3	17.1				
3	43.8	20.0		33.1	31.3		48.8	38.8	17.5	22.9				
4	16.3	1.3		8.8	9.3		16.3	16.3	1.3	1.4				
HB^2^	0.44^a^	0.06^b^	0.04	0.21	0.31	0.05	0.41	0.48	0.01	0.11	0.06	<0.001	0.11	0.16
0	69.4	95.3		83.8	80.0		68.8	70.0	98.8	91.4				
1	19.4	3.3		12.5	10.7		23.8	15.0	1.3	5.7				
2	8.8	1.3		2.5	8.0		5.0	12.5	0	2.9				
3	2.5	0		1.3	1.3		2.5	2.5	0	0				
4	0	0		0	0		0	0	0	0				

a-b Per factor, values in a row lacking a common superscript differ significantly (*P* < 0.05).

1 GS = gait score, where score 0 = walking perfectly and 5 = unable to walk

2 FPD = footpad dermatitis and HB = hock burns, where score 0 = no lesion and 4 = severe lesion. Abbreviations: ADAP, adapted treatment (i.e., organic macro minerals and a platform); CON, control (i.e., inorganic macro minerals without enrichment); FAST, fast-growing broilers; SLOW, slower-growing broilers; (n = 8 pens for FAST-CON, FAST-ADAP and SLOW-CON, n = 7 pens for SLOW-ADAP).

### Leg Disorders and Breast Myopathies

No significant effects of the interaction between breed and treatment or treatment alone were found for leg disorders (EPA, EPI, and VV) and WB. Breed effects were found, with FAST broilers having lower EPA, and higher VV (overall, left, and right leg) and WB than SLOW broilers at 2.3 kg (Table 6).

**Table 6:** Effects of breed, treatment, and their interaction on leg disorders and breast myopathies, values represent means and percentages of birds per score are given.

Variable	Breed			Treatment			Interaction					*P*-values		
	FAST	SLOW	SEM	CON	ADAP	SEM	FAST-CON	FAST-ADAP	SLOW-CON	SLOW-ADAP	SEM	Breed	Treatment	Breed*Treatment
EPA^1^	0.03^b^	0.22^a^	0.02	0.13	0.11	0.03	0.03	0.04	0.24	0.20	0.03	<0.001	0.83	0.51
0	96.9	78.0		86.9	88.7		97.5	96.3	76.3	80.0				
1	3.1	22.0		13.1	11.3		2.5	3.8	23.8	20.0				
EPI^1^	0.18	0.14	0.03	0.19	0.13	0.03	0.24	0.11	0.14	0.14	0.04	0.65	0.25	0.22
0	82.5	86.0		81.3	87.3		76.3	88.8	86.3	85.7				
1	17.5	14.0		18.8	12.7		23.8	11.3	13.8	14.3				
TD^1^	0.01	0.01	0.01	0.01	0	0.01	0.01	0	0.01	0	0.01			
0	99.4	99.3		98.8	100.0		98.8	100.0	98.8	100.0				
1	0.6	0.7		1.3	0		1.3	0	1.3	0				
BCO^1^	0.02	0.03	0.01	0.03	0.01	0.01	0.04	0	0.03	0.03	0.02			
0	98.1	97.3		96.9	98.7		96.3	100.0	97.5	97.1				
1	1.9	2.7		3.1	1.3		3.8	0	2.5	2.9				
VV^2^	4.4^a^	2.8^b^	0.2	3.5	3.6	0.2	4.2	4.6	2.9	2.7	0.3	<0.001	0.71	0.39
VV^2^ (left)	3.8^a^	2.9^b^	0.2	3.3	3.4	0.2	3.6	3.9	3.0	2.8	0.3	0.006	0.98	0.35
VV^2^ (right)	5.0^a^	2.6^b^	0.3	3.7	3.9	0.3	4.8	5.3	2.7	2.6	0.4	<0.001	0.56	0.48
WB^3^	0.33^a^	0.07^b^	0.03	0.19	0.21	0.04	0.30	0.35	0.08	0.06	0.05	0.002	0.87	0.37
0	75.0	93.3		85.6	82.0		78.8	71.3	92.5	94.3				
1	17.5	6.7		10.0	14.7		12.5	22.5	7.5	5.7				
2	7.5	0		4.4	3.3		8.8	6.3	0	0				
3	0	0		0	0		0	0	0	0				
WS^3^	0.10	0.01	0.02	0.05	0.07	0.02	0.10	0.10	0	0.03	0.03			
0	91.3	98.7		95.6	94.0		91.3	91.3	100.0	97.1				
1	7.5	1.3		3.8	5.3		7.5	7.5	0	2.9				
2	1.3	0		0.6	0.7		1.3	1.3	0	0				
3	0	0		0	0		0	0	0	0				

a-b Per factor, values in a row lacking a common superscript differ significantly (*P* < 0.05).

1 EPA = epiphyseal plate abnormalities, EPI = epiphysiolysis, TD = tibial dyschondroplasia and BCO = bacterial chondronecrosis with osteomyelitis, where 0 = no abnormalities and 1 = abnormalities.

2 VV = varus valgus in °.

3 WB = wooden breast and WS = white striping, where 0 = normal and 3 = extreme. Abbreviations: ADAP, adapted treatment (i.e., organic macro minerals and a platform); CON, control (i.e., inorganic macro minerals without enrichment); FAST, fast-growing broilers; SLOW, slower-growing broilers; (n = 8 pens for FAST-CON, FAST-ADAP, and SLOW-CON, n = 7 pens for SLOW-ADAP).

### Tibia Characteristics

No significant effects of the interaction between breed and treatment were found for tibia characteristics. Breed effects were found, with FAST broilers having lower tibia weight, length, pore volume, mineral content and metatarsal head thickness, and higher tibia osseous volume, volume fraction and elastic modulus than SLOW broilers. Furthermore, treatment effects were found, with broilers in CON treatment having lower tibia osseous volume, total volume and mineral density than broilers in ADAP treatment at slaughter weight (Table 7).

**Table 7:** Effects of breed, treatment, and their interaction on tibia characteristics.

Variable	Breed			Treatment			Interaction					*P*-values		
	FAST	SLOW	SEM	CON	ADAP	SEM	FAST-CON	FAST-ADAP	SLOW-CON	SLOW-ADAP	SEM	Breed	Treatment	Breed*Treatment
Morphological characteristics														
Tibia weight (g)	10.3^b^	11.5^a^	0.1	10.9	10.9	0.1	10.2	10.3	11.5	11.5	0.1	**<0.001**	0.93	0.74
Tibia length (cm)	9.79^b^	10.84^a^	0.06	10.35	10.28	0.06	9.86	9.71	10.84	10.84	0.08	**<0.001**	0.37	0.37
Tibia thickness (cm)	1.59	1.50	0.04	1.50	1.60	0.04	1.50	1.69	1.50	1.51	0.06	0.17	0.12	0.18
Femoral head thickness (cm)	3.77	3.70	0.08	3.68	3.79	0.08	3.66	3.88	3.70	3.70	0.10	0.45	0.23	0.22
Metatarsal head thickness (cm)	3.42^b^	3.55^a^	0.04	3.48	3.50	0.04	3.40	3.45	3.56	3.54	0.06	**0.04**	0.80	0.60
Robusticity index (cm/g)	0.97	0.96	0.01	0.97	0.96	0.01	0.98	0.96	0.96	0.97	0.01	0.63	0.74	0.29
Biophysical characteristics														
Tibia osseous volume (cm^3^)	21.8^a^	20.3^b^	0.2	20.1^b^	22.0^a^	0.2	20.8	22.7	19.4	21.3	0.3	**<0.001**	**<0.001**	0.94
Tibia pore volume (cm^3^)	3.2^b^	4.7^a^	0.3	4.0	3.8	0.3	3.1	3.3	4.9	4.4	0.4	**<0.001**	0.62	0.42
Tibia total volume (cm^3^)	25.0	25.0	0.4	24.1^b^	25.8^a^	0.4	23.9	26.0	24.3	25.7	0.6	1.0	**0.008**	0.58
Tibia volume fraction (OV/TV, %)^1^	87.6^a^	81.8^b^	1.0	83.9	85.5	1.0	87.3	87.8	80.5	83.1	1.2	**<0.001**	0.18	0.37
Tibia mineral content (g)	9.4^b^	9.8^a^	0.1	9.6	9.5	0.1	9.5	9.2	9.8	9.8	0.2	**0.02**	0.32	0.34
Tibia mineral density (g/cm^3^)	0.27	0.28	0.01	0.25^b^	0.30^a^	0.01	0.25	0.29	0.25	0.32	0.01	0.10	**<0.001**	0.15
Mechanical characteristics														
Ultimate strength (N)	269	258	6	264	263	6	275	263	253	262	9	0.23	0.86	0.23
Stiffness (N/mm)	197	191	8	193	195	8	202	192	184	199	12	0.62	0.84	0.28
Energy to fracture (N-mm)	223	212	9	219	216	9	231	216	207	217	12	0.36	0.85	0.31
Elastic modulus (GPa)	10.2	5.3	0.6	8.3	7.1	0.6	10.9	9.4	5.6	4.9	0.8	**<0.001**	0.12	0.59

a-b Per factor, values in a row lacking a common superscript differ significantly (*P* < 0.05).

1 OV = osseous volume and TV = total volume. Abbreviations: ADAP, adapted treatment (i.e., organic macro minerals and a platform); CON, control (i.e., inorganic macro minerals without enrichment); FAST, fast-growing broilers; SLOW, slower-growing broilers; (n = 8 pens for FAST-CON, FAST-ADAP, and SLOW-CON, n = 7 pens for SLOW-ADAP).

## DISCUSSION

The aim of this study was to identify the combined effect of replacing inorganic with organic macro minerals and providing an elevated platform on leg health, tibia characteristics, behavior and performance in fast- and slower-growing broilers. We hypothesized that the adapted treatment would have a positive effect on leg health and tibia characteristics in both fast- and slower-growing broilers. Fast- and slower-growing broilers did not differ in their response to the control and adapted treatment with regard to leg health, tibia characteristics, behavior and performance. Overall, slower-growing broilers had a better walking ability and tibia characteristics, less contact dermatitis, showed more locomotion and foraging behavior, less ingestion behavior and had lower performance compared to fast-growing broilers. The adapted treatment did not affect leg health, morphological and mechanical tibia characteristics, and improved biophysical tibia characteristics, and increased locomotion and performance.

### Leg Health

Slower-growing broilers had better gait scores, and less footpad dermatitis and hock burn compared to fast-growing broilers just prior to 2.3 kg. These findings are supported by previous studies (Kjaer et al., 2006; Dixon, 2020; Rayner et al., 2020; Güz et al., 2021a), although differences between breeds are not always found (de Jong et al., 2021). At 2.3 kg, slower-growing broilers had more epiphyseal plate abnormalities and lower varus valgus compared to fast-growing broilers, and no differences were found for epiphysiolysis. It is known that gait score is not always correlated with leg abnormalities or disorders (Sandilands et al., 2011), which might explain our contradicting results for gait score and leg disorders. Furthermore, it should be noted that the prevalence of leg disorders was low in the current study. Previous studies also found lower varus valgus when comparing slower- to fast-growing broilers (Güz et al., 2021a) or broilers from the same breed with slow or fast growth rates (Shim et al., 2012a). However, despite the difference between breeds, the maximum average angulation in the current study was 5.03° and it can be disputed whether this degree of angulation can be considered as varus valgus or as a leg disorder. Overall, slower-growing broilers had better walking ability and less contact dermatitis, which might be caused by their higher activity level and better use of enrichments as discussed later on. Yet, with regard to leg disorders results were less consistent, which might be related to the low prevalence of leg disorders in the current study.

No effects of treatment were found on leg disorders, contact dermatitis or walking ability. Most studies support these findings, where providing environmental enrichments or replacing inorganic by organic minerals did not affect the prevalence of leg disorders, contact dermatitis or walking ability for fast- and slower-growing broilers (Kaukonen et al., 2017b; Bailie et al., 2018; de Jong and Gunnink, 2019; Güz et al., 2019, 2021b,a; Pedersen et al., 2020; Tahamtani et al., 2020; de Jong et al., 2021; Malchow and Schrader, 2021). Although some reported better gait score, and lower incidence of contact dermatitis or tibial dyschondroplasia in fast-growing chickens (Zhao et al., 2013; Kaukonen et al., 2017a), and better or worse footpad health in dual-purpose chickens (Malchow et al., 2019; Malchow and Schrader, 2021) when providing elevated structures. Well-known risk factors for contact dermatitis are poor litter quality and long periods of contact with the litter due to inactivity (Bessei, 2006). In the current study, treatments did not differ in litter quality or inactive behavior, and although broilers in the adapted treatment showed more locomotion at 1.9 kg, it can be questioned whether a difference of 1.39% is large enough to influence contact dermatitis or walking ability. Since it has been suggested that increasing locomotor activity of broilers might improve leg health (Kestin et al., 1992; Prayitno et al., 1997a,b; Reiter and Bessei, 2009; Stojcic and Bessei, 2009). Overall, the adapted treatment did not affect leg health in both fast- and slower-growing broilers, although it should be noted that the prevalence of leg disorders was low in the current study.

### Tibia Characteristics

Slower-growing broilers had higher tibia weight, length, pore volume, mineral content and metatarsal head thickness, and lower osseous volume, volume fraction and elastic modulus than fast-growing broilers at 2.3 kg. Previously, broilers with slow growth rates had lower tibia weight, length, mineral density, mineral content and breaking strength compared to broilers with fast growth rates from the same breed (Shim et al., 2012b). This discrepancy with our study is likely explained by sampling at the same age instead of at similar body weight. Our findings are supported by Güz et al. (2021a), who also sampled at similar body weight, although they found the opposite for tibia osseous volume, total volume, and volume fraction. Furthermore, mechanical characteristics were higher for slower-growing broilers compared to fast-growing broilers and breeds did not differ for elastic modulus (Güz et al., 2021a). Previous studies have also indicated that slower-growing broilers demonstrate better bone characteristics compared to fast-growing broilers (Leterrier and Nys, 1992; Williams et al., 2000, 2004). Overall, slower-growing broilers had better tibia characteristics, likely resulting in improved leg health, although it should be noted that for biophysical and mechanical characteristics results are not always consistent.

The adapted treatment improved biophysical characteristics (tibia osseous volume, total volume, mineral density) compared to the control treatment, but did not affect morphological or mechanical characteristics. These findings are supported by previous studies, where providing environmental enrichments or replacing inorganic by organic macro minerals did not affect morphological or mechanical characteristics (Guinotte et al., 1991; Pedersen et al., 2020; Güz et al., 2021a), and improved biophysical characteristics (Güz et al., 2021b, a), suggesting both the organic mineral diet and enrichment might contribute to the positive effects, especially on biophysical characteristics. Yet, results are inconclusive as one study found no effect of providing environmental enrichments on bone mineral content (Kaukonen et al., 2017a) and replacing inorganic by organic macro minerals was found to improve mechanical characteristics (Güz et al., 2021b). Adding a platform to the treatment might have limited the positive effects of organic macro minerals on mechanical characteristics, as previously environmental enrichments did not affect mechanical characteristics (Pedersen et al., 2020; Güz et al., 2021a). Yet, it remains unclear what causes the differences in effects. Overall, the adapted treatment did not affect morphological or mechanical tibia characteristics and improved biophysical characteristics.

### Behavior

Overall, slower-growing broilers showed less ingestion behavior and more locomotion, foraging and aggressive behavior at 0.6 kg and more locomotion at 1.9 kg compared to fast-growing broilers, which is supported by previous studies (Bokkers and Koene, 2003; Wallenbeck et al., 2016; Dixon, 2020; Rayner et al., 2020; de Jong et al., 2021; Güz et al., 2021a). Interestingly, it is often suggested that differences in time budgets are caused by differences in growth rate or body weight (Bokkers and Koene, 2003; Wallenbeck et al., 2016). Yet, in this study we compared fast- and slower-growing broilers at a similar body weight, as was also done by de Jong et al. (2021) and Güz et al. (2021a). Therefore, differences in behavior might be more related to genetic background rather than body weight. Fast-growing broilers showed less locomotion at 1.9 kg, which might be related to the presence of leg problems and wooden breast, since leg problems may cause pain or physical limitations (Weeks et al., 2000) and wooden breast affected birds had a worse walking ability (Norring et al., 2019). In the present study, fast-growing broilers had worse gait scores and a higher prevalence of contact dermatitis and wooden breast compared to slower-growing broilers at similar body weight. Overall, slower-growing broilers showed more locomotion, foraging and aggressive behavior and less ingestion, which is most likely caused by their genetic background and might further be related to their better leg health (walking ability and contact dermatitis) increasing the performance of locomotive behaviors.

Broilers in the adapted treatment showed more locomotion compared to broilers in the control treatment at 1.9 kg. These differences might have mainly been caused by the enrichments provided, as previous studies showed that receiving an organic mineral diet did not affect home pen behavior in fast-growing broilers compared to an inorganic mineral diet (Güz et al., 2019, 2021b). Indeed, it has been suggested that providing elevated structures (i.e., perches or platforms) might stimulate a greater variety of locomotor activities (Bizeray et al., 2002; Kaukonen et al., 2017a). However, previous studies found no increase in general locomotor activity when elevated structures were provided (Bizeray et al., 2002; Bailie and O'Connell, 2015; Norring et al., 2016; Bach et al., 2019; Baxter et al., 2019, 2020), with the exception of one study (Malchow et al., 2019). The increase in locomotion might have been caused by broilers having a better walking ability, as lame birds tend to be less active (Weeks et al., 2000). However, in the present study we found no effect of treatment on leg health (including walking ability). Overall, the adapted treatment increased locomotion, but it appears this was not sufficient to improve leg health.

### Enrichment Use

At both 0.6 and 1.9 kg, slower-growing broilers were observed more on the platform and ramps compared to fast-growing broilers. At 1.9 kg, fast-growing broilers were observed more underneath the platform and ramps. These findings are supported by previous studies where slower-growing broilers made more use of elevated resting structures (i.e., platforms and perches) compared to fast-growing broilers (Bokkers and Koene, 2003; Wallenbeck et al., 2016; Malchow et al., 2019; de Jong et al., 2021; Güz et al., 2021a; Malchow and Schrader, 2021), although no difference was found between breeds for percentage of broilers underneath a platform and ramps (de Jong et al., 2021). The higher use of platforms by slower-growing broilers could be caused by their higher activity level in general (Bokkers and Koene, 2003; Wallenbeck et al., 2016; Dixon, 2020; de Jong et al., 2021), as was also found in the present study, potentially resulting in more slower-growing broilers climbing the ramp. It could also be caused by slower-growing broilers having a different body conformation compared to fast-growing broilers (Norring et al., 2016; de Jong and Gunnink, 2019), resulting in fewer problems with finding their balance or in a better ability to fly, walk or climb on the platform.

### Performance

As expected, slower-growing broilers had a lower body weight, slaughter weight, daily feed intake, and a higher feed conversion ratio compared to fast-growing broilers, which is supported by previous studies (Dixon, 2020; Rayner et al., 2020; de Jong et al., 2021; Güz et al., 2021a). The lower mortality for slower-growing broilers compared to fast-growing broilers was also found by Dixon (2020) and Rayner et al. (2020), but not in de Jong et al. (2021). In addition, slower-growing broilers had lower prevalence of wooden breast compared to fast-growing broilers, which is supported by previous studies (Dixon, 2020; Santos et al., 2021). Overall, slower-growing broilers showed lower performance, mortality and prevalence of wooden breast compared to fast-growing broilers.

Broilers in the adapted treatment were found to have a higher body weight, slaughter weight, daily feed intake, and a lower feed conversion ratio compared to broilers in the control treatment. Previous studies support these findings where fast-growing broilers receiving an organic mineral diet showed higher body weight gain and lower feed conversion ratio (Bradbury et al., 2018; Güz et al., 2019, 2021b), while another study reported no effect on body weight gain and feed conversion ratio (Bradbury et al., 2017). Previous studies where platforms were provided reported no effect on performance of fast- and slower-growing broilers (Bailie et al., 2018; Baxter et al., 2020; Jones et al., 2020; Malchow and Schrader, 2021), or even a negative effect when platforms were combined with other enrichments (de Jong et al., 2021; Güz et al., 2021a). No effects of treatment on wooden breast were found, which is supported by a previous study where providing an elevated platform did not affect wooden breast (Pedersen et al., 2020). Although we cannot separate the effects of organic macro minerals and enrichment provided in the present study, findings from previous studies suggest that the positive effect on performance might have mainly been caused by replacement of inorganic by organic macro minerals. Overall, the adapted treatment increased performance, in terms of body weight and feed conversion ratio, of both fast- and slower-growing broilers.

## CONCLUSION

In conclusion, fast- and slower-growing broilers responded to the treatment similarly. The adapted treatment (i.e., organic macro minerals and a platform) improved biophysical tibia characteristics, and increased locomotion and performance, but did not affect leg health, morphological and mechanical tibia characteristics. These findings indicate that the adapted treatment could improve leg health in both fast- and slower-growing broilers. However, in the current study, these positive effects on tibia characteristics and locomotion seem to be insufficient to improve leg health, which might be related to the low prevalence of leg disorders.
